# Non-Arteritic Anterior Ischemic Optic Neuropathy (NA-AION) and High-Risk Cardiovascular Events

**DOI:** 10.3390/life16091401

**Published:** 2026-08-24

**Authors:** Christiana-Diana-Maria Dragosloveanu, Vasile Potop, Alina-Gabriela Gheorghe, Tudor-George Potop, Maria-Cristina Marinescu, Ana Maria Arghirescu, Ioana-Maria Rizea, Dana-Margareta-Cornelia Dăscălescu

**Affiliations:** 1 Department of Ophthalmology, Carol Davila University of Medicine and Pharmacy, 020021 Bucharest, Romania; christiana.dragosloveanu@umfcd.ro (C.-D.-M.D.); vasile.potop@umfcd.ro (V.P.); dana.dascalescu@umfcd.ro (D.-M.-C.D.); 2 Doctoral School, Carol Davila University of Medicine and Pharmacy, 020021 Bucharest, Romania; ana-maria.arghirescu1025@rez.umfcd.ro (A.M.A.); ioana-maria.vlad@drd.umfcd.ro (I.-M.R.); 3 Department of Ophthalmology, Clinical Institute of Ophthalmological Emergencies “Prof. Dr. Mircea Olteanu”, 010464 Bucharest, Romania; 4 Medical Physiology Discipline, Carol Davila University of Medicine and Pharmacy, 020021 Bucharest, Romania; maria.marinescu@umfcd.ro

**Keywords:** anterior ischemic optic neuropathy, NA-AION, atheromatosis, visual field defects, hypoperfusion

## Abstract

Background: Anterior ischemic optic neuropathy (AION) is a potentially vision threatening disorder that affects middle-aged and elderly individuals. The pathogenesis of the disease remains incompletely understood. However, the most universally accepted hypothesis suggests that a transient reduction in optic nerve head (ONH) perfusion, secondary to hypovolemia, nocturnal hypotension, or small-vessel disease, results in ischemic infarction of the anterior portion of the optic nerve. Case presentation: We present the case of a 61-year-old male patient with a history of arterial hypertension, type II diabetes mellitus, generalized atheromatosis, femoral bypass and stent implantation for chronic ischemia in his left leg, followed by brachial artery dissection that needed a prompt vascular intervention; he presented visual acuity loss in both eyes (BE), with the left eye (LE) being more affected than the right eye (RE). A complete ophthalmologic check-up was performed, and it revealed best-corrected visual acuity (BCVA) RE 0.5 Snellen, LE light perception, normal intraocular pressure (IOP), BE papillary edema and arteriovenous abnormalities. Visual field (VF) examination revealed RE inferior altitudinal defect and LE preabsolute scotoma. The diagnosis of BE anterior ischemic optic neuropathy and hypertensive retinopathy stage II was formulated. Following the aforementioned surgical procedures meant optimizing the patient’s critical vascular status, subsequent management of NA-AION systemic risk factors, along with implementing important lifestyle changes. No further cardiovascular events occurred, and visual field parameters remained stable at the three-year follow-up. Conclusions: Non-arteritic anterior ischemic optic neuropathy (NA-AION) remains a topic of interest for both ophthalmologists and neurologists worldwide. When confronted with a high-risk cardiovascular patient, a rapid and thorough systemic evaluation may prove beneficial. This management strategy could potentially contribute to maintaining a stable systemic outcome, despite major cardiovascular threats.

## 1. Introduction

Ischemic optic neuropathy (ION) is a sight-threatening condition with complex etiology, frequently associating structural crowding of the optic nerve head, hypoperfusion and cardiovascular risk factors [1].

From a clinical perspective, ischemic optic neuropathies may be classified into anterior ischemic optic neuropathy (AION) and posterior ischemic optic neuropathy (PION). AION is characterized by edema of the optic disc, whereas PION does not associate optic disc swelling [2]. In addition, AION is further categorized as arteritic (A-AION), when associated with an underlying arteritic process or non-arteritic/idiopathic neuropathy (NA-AION). In NA-AION, a small or absent physiological optic cup, commonly referred to as a “disc at risk”, is considered an important predisposing anatomical feature, alongside optic nerve head hypoperfusion and systemic vascular risk factors [3,4]. However, the etiology and pathophysiology of these conditions remain incompletely understood and continue to be debated.

The incidence of AION has been estimated to be 2–10 new cases per 100,000 people per year, while the prevalence of the disease has been estimated at approximately 3–10 cases per 100,000 people [5]. In Caucasian populations, NA-AION is considered the most common acute optic neuropathy in adults over 50 years of age, having an estimated incidence of approximately 2.3–10.2 cases per 100,000 people per year [6]. The disease was first described by French physician Jean-Pierre Saint-Yves in 1817. Later, in 1935, the clinical picture of NA-AION was described by C. Miller Fisher [7].

The pathological process underlying NA-AION is thought to occur as a consequence of impaired perfusion within the short posterior ciliary arteries, which supply the ONH. The optic nerve head is primarily vascularized by branches of the ophthalmic artery itself, a terminal branch of the internal carotid artery. Among these branches, the short posterior ciliary arteries play a key role in supplying the prelaminar and laminar regions of the ONH [8].

Histologic studies revealed an area of infarction located within the scleral canal, suggesting the mechanism of a potential local compartment syndrome. This leads to tissue hypoxia and disruption of axoplasmic transport, resulting in ONH swelling during the acute stage. This process is clinically seen as optic disc swelling. In addition to this, the clinical picture of NA-AION is also defined by a sudden, painless decrease in visual acuity, impaired color perception, the presence of relative afferent pupillary defect (RAPD), and, in some cases, flame-shaped hemorrhages on or close to the optic disc. In time, as the acute phase passes, the swelling gradually diminishes and the clinical appearance of the optic disc changes from edema to pallor or atrophy, accompanied by permanent VF defects due to irreversible axonal loss and retinal ganglion cell damage [9].

Several precipitating factors have been involved as possible triggers of the pathological mechanisms observed in NA-AION. These include nocturnal hypotension, systemic hypoperfusion, vasospasm, intraoperative fluid shifts, hemodilution, local arteriosclerotic vascular changes, embolic events, venous occlusion, thrombosis and multiple microemboli originating from a distant source [6,7,10]. Additionally, several local risk factors may contribute. Hyperopic patients, with normal IOP and smaller axial length that are associated with a structurally crowded optic disc, characterized by a small optic disc with a reduced or absent physiological cup—the so-called “disc at risk”—are particularly susceptible to this compartment-like phenomenon [3,11,12].

The disc at risk (defined as a small optic disc diameter < 1.5 mm, with a c/d < 0.2) is currently thought to be a possible predisposing anatomical substrate rather than a predictive marker [7]. In the largest available unilateral NA-AION case–control series, comparing 1812 unaffected fellow eyes of patients with NA-AION against control eyes, the odds of NA-AION were inversely proportional to cup-to-disc ratio (CDR): eyes with a CDR ≤ 0.20 showed approximately 46-fold greater odds than eyes with a CDR > 0.40 [13]. Neither threshold, however, performs adequately as a test: a CDR ≤ 0.20 was found in 54% of fellow eyes but also in 9% of control eyes, and a CDR ≤ 0.40 in 94% versus 56%, respectively [13]. Consequently, the high prevalence of a small CDR in the general population relative to the incidence of the disease (3.89–10.19 per 100,000 individuals) precludes its use as an individual predictive marker [14].

In order to prevent visual loss and particularly to preserve the fellow eye, it is essential to have an early accurate diagnosis. Therefore, an extensive approach that includes systemic evaluation, risk factors assessment and interdisciplinary work-up is required in order to save vision and improve outcomes in patients with this potentially vision-threatening condition [15].

The most frequent presentation of NA-AION is unilateral [6]. Although NA-AION is a well-established clinical entity, simultaneous bilateral involvement remains uncommon [16]. Studies associate these rare NA-AION cases with extreme bleeding, hemodialysis and anemia [17,18]. The present paper presents a bilateral simultaneous NA-AION in a high-risk cardiovascular patient, highlighting the cumulative effect of impaired oxygen delivery, reduced systemic perfusion, and advanced vascular disease, as well as the diagnostic challenges in the setting of successive major vascular surgeries.

## 2. Case Presentation

We present the case of a 61-year-old male diabetic patient, smoker, with generalized atheromatosis, who reported sudden painless visual acuity loss in both eyes (BE), with the LE being more affected than the RE. Patient history revealed 54% stenosis of the internal carotid arteries with extended calcifications, 95% left external iliac artery and 80% left femoral artery stenosis. The patient underwent a left femoral bypass and stent implantation in the external iliac artery for chronic ischemia in his left leg. Soon after, he suffered a brachial artery dissection that needed emergency vascular surgery.

Visual acuity loss developed five days after discharge. The brachial artery dissection had been surgically repaired and a femoral bypass performed; at the time of the ophthalmic presentation, the patient was hemodynamically stable. He was submitted to a complete ophthalmologic consult, including best-corrected visual acuity (BCVA), Goldmann applanation for IOP measurement, Humphrey VF testing (SITA Standard 30-2) [19], and optical coherence tomography (OCT) for the optic nerve head and macular region.

The clinical examination revealed a BCVA of 20/40 Snellen in the RE and light perception (LP) in the LE. IOP was 14 mmHg in the RE and 16 mmHg in the LE. Slit-lamp examination of the anterior segment (AS) did not reveal any AS structural abnormalities that could account for the visual acuity loss. Apart from a relative afferent pupillary defect (RAPD), detected in the left eye, which indicated an asymmetric optic nerve dysfunction, no other anterior segment signs were noted in either eye. Fundus examination in both eyes revealed a swollen and hyperemic optic disc, arteriolar narrowing, turgescent veins and arteriovenous crossing abnormalities.

**Figure 1:**
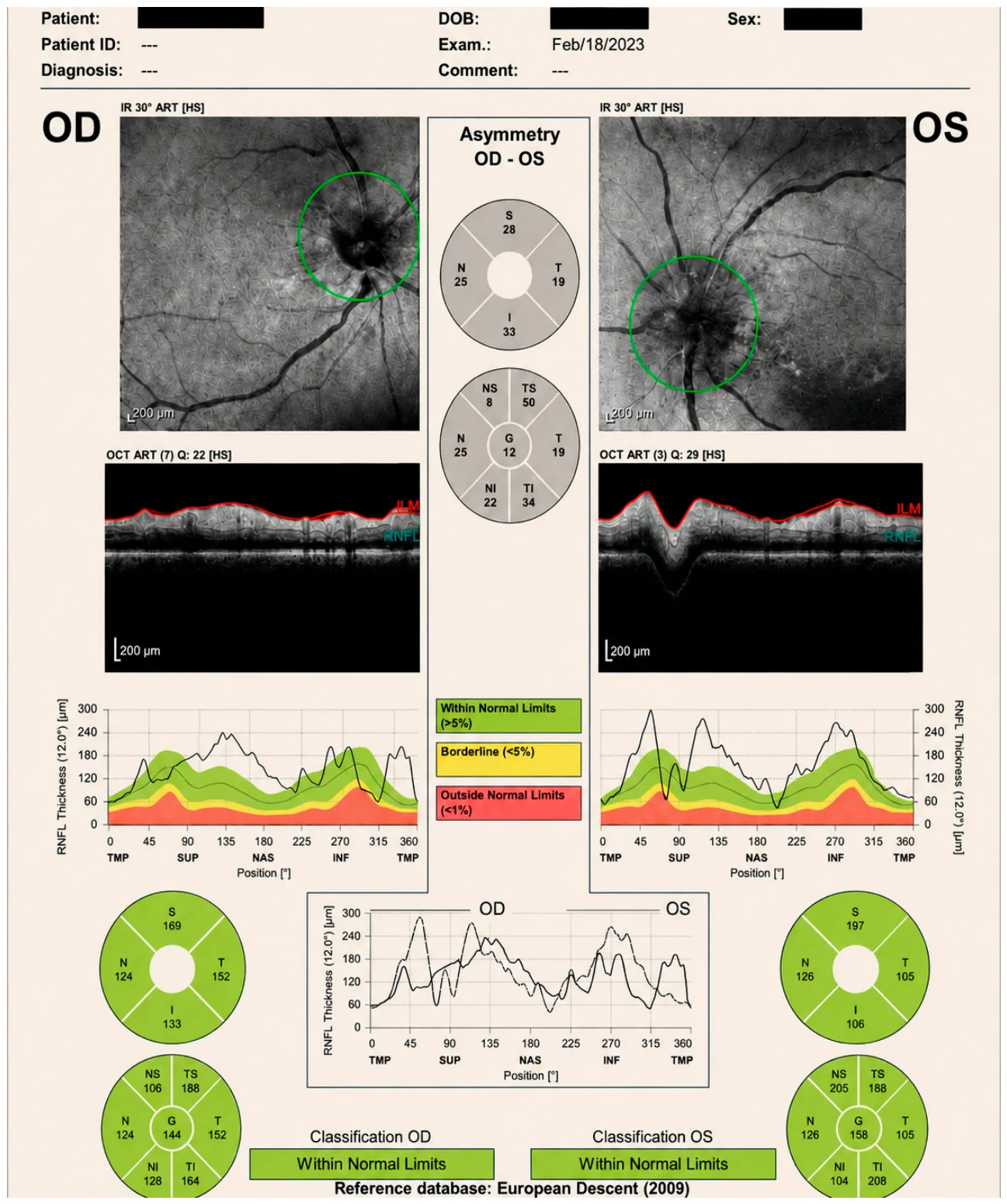
OCT showing BE optic disk edema LE > RE.

**Figure 2:**
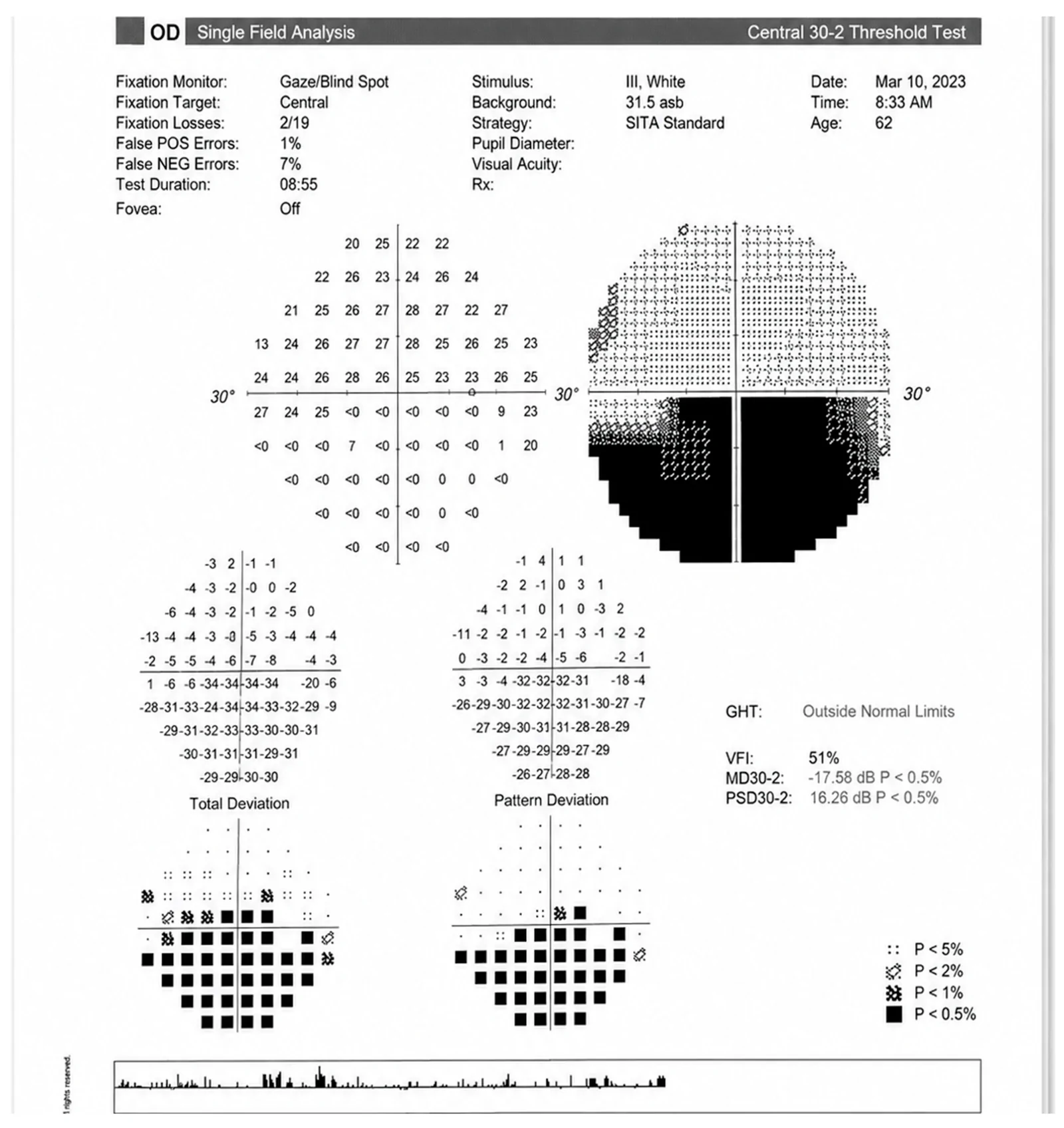
Humphrey VF at first presentation RE—inferior altitudinal defect.

**Figure 3:**
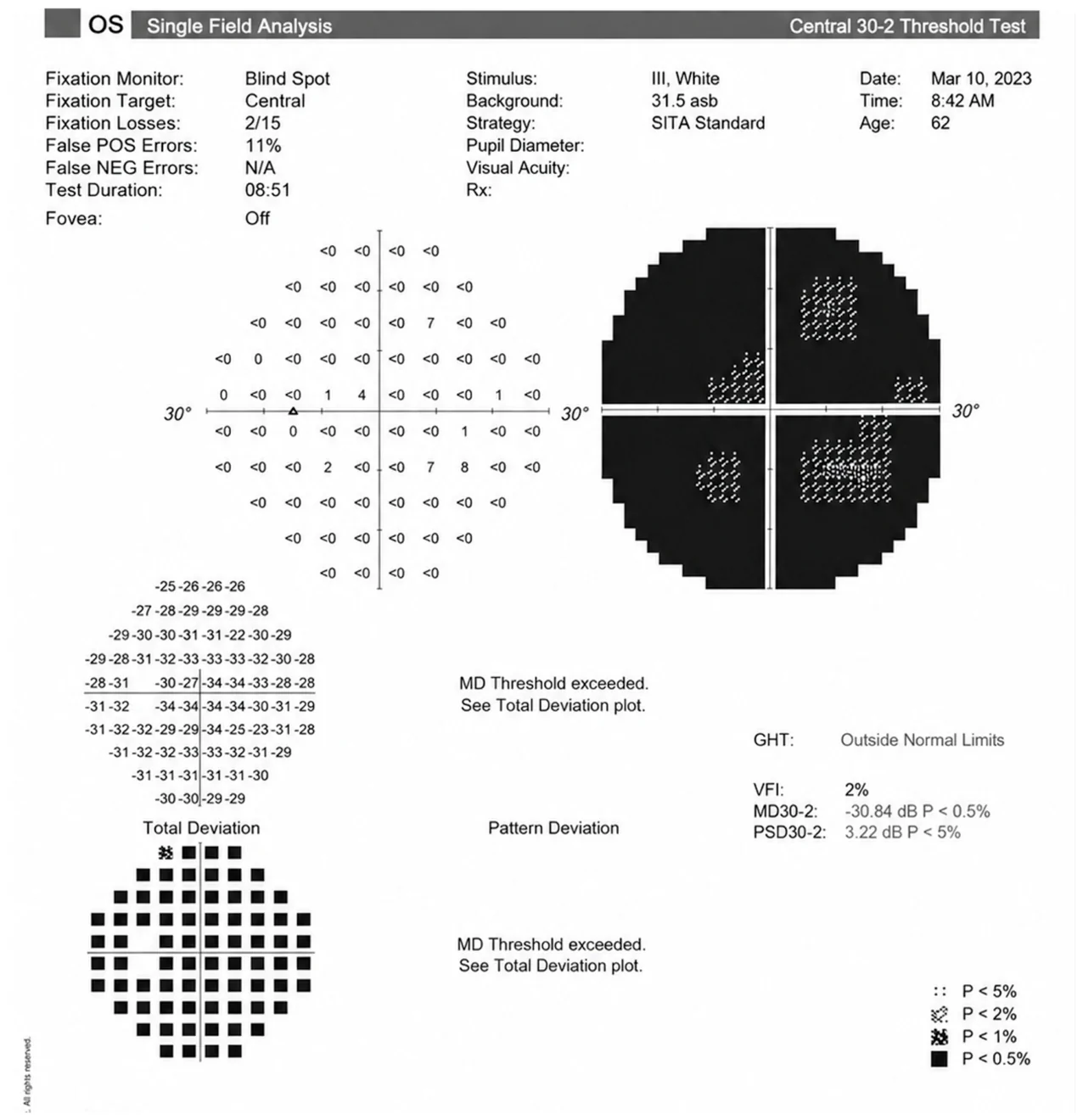
Humphrey VF at first presentation LE—preabsolute scotoma.

OCT findings showed increased retinal nerve fiber layer (RNFL) thickness LE > RE (Figure 1). Macular OCT showed no significant abnormalities. Standard automated perimetry detected an inferior altitudinal defect in the right eye (Figure 2) and a preabsolute scotoma in the left eye (Figure 3) corresponding to the OCT findings. Also, from a clinical point of view, the patient perceived the altitudinal defect from the RE.

A comprehensive systemic work-up revealed severe anemia with a hemoglobin level of 6.9 g/dL, hematocrit (Hct) of 20.6%, and mean corpuscular volume (MCV) of 92 fL, consistent with normocytic anemia, coupled with low platelets count and a blood glucose level of 117 mg/dL (Table 1). In addition, the laboratory findings were remarkable for mildly elevated inflammatory markers, both C-reactive protein and erythrocyte sedimentation rate (ESR). The acute-phase markers were measured serially from the initial ophthalmological presentation onward. The CRP normalized within 7 days and the ESR fell from 45 to 27 mm/h over the same interval, reaching normal values by 3 weeks. This occurred entirely without immunosuppressive treatment; thus, the initial rise was probably due to the increased surgical stress. Arterial blood pressure was 90/50 mmHg and heartbeat rate 116 beats/minute, also probably due to the systemic vascular status of the patient.

The patient underwent a computed tomograph (CT) angiography of the supra-aortic trunks, which revealed atheromatosis and a 54% stenosis of the internal carotid arteries, with extended calcifications especially on the left side. The coagulation panel revealed a fibrinogen value of 713 mg/dL and a D-dimer level of 331 ng/mL. Laboratory findings revealed impaired renal function (serum creatinine 1.52 mg/dL, GFR 51 mL/min/1.73 m^2^), with a disproportionately elevated urea (87.74 mg/dL; BUN/creatinine ratio 27). These indicated a mild, residual prerenal state related to perioperative stress. Magnetic resonance imaging (MRI) of the brain and vessels showed some mild ischemic microlesions, without mural inflammation in the superficial temporal arteries.

The differential diagnosis included NA-AION, AION, papillitis, papilledema, central retinal artery occlusion, central retinal vein occlusion and optic nerve head drusen. The most complex differential diagnosis was with AION, but the patient reported no headache, no jaw claudication, and no scalp tenderness, and the MRI showed no specific signs of inflammation in the superficial temporal arteries; a temporal artery biopsy was not possible due to the high surgical risk.

Based on the ophthalmologic examination, comprehensive systemic evaluation, paraclinical findings, relevant patient history and head vessels MRI, a final diagnosis of bilateral NA-AION and hypertensive retinopathy stage II was established.

After the management of the major cardiovascular event and once hemodynamic stability was fully achieved, the long-term treatment included systemic antiaggregant and anticoagulant that was associated with blood-sugar-lowering medication, lipid-lowering hypotensive agents, and vasodilators. Smoking was discouraged, while diet and regular physical activity were started.

**Figure 4:**
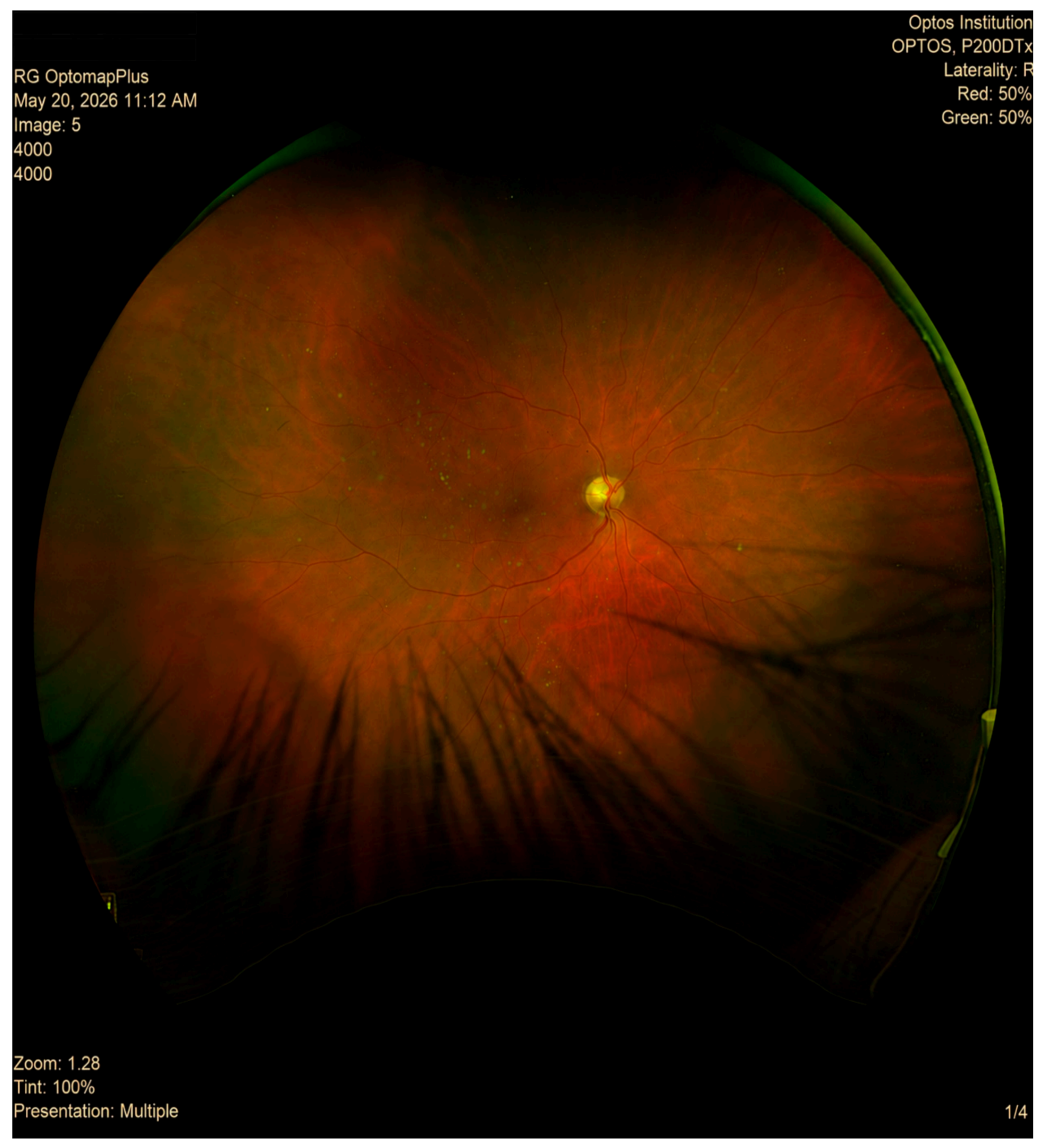
Patient’s fundus photo at 3 years follow-up: RE pale disc in the superior part, turgescent veins and arteriovenous crossing abnormalities.

**Figure 5:**
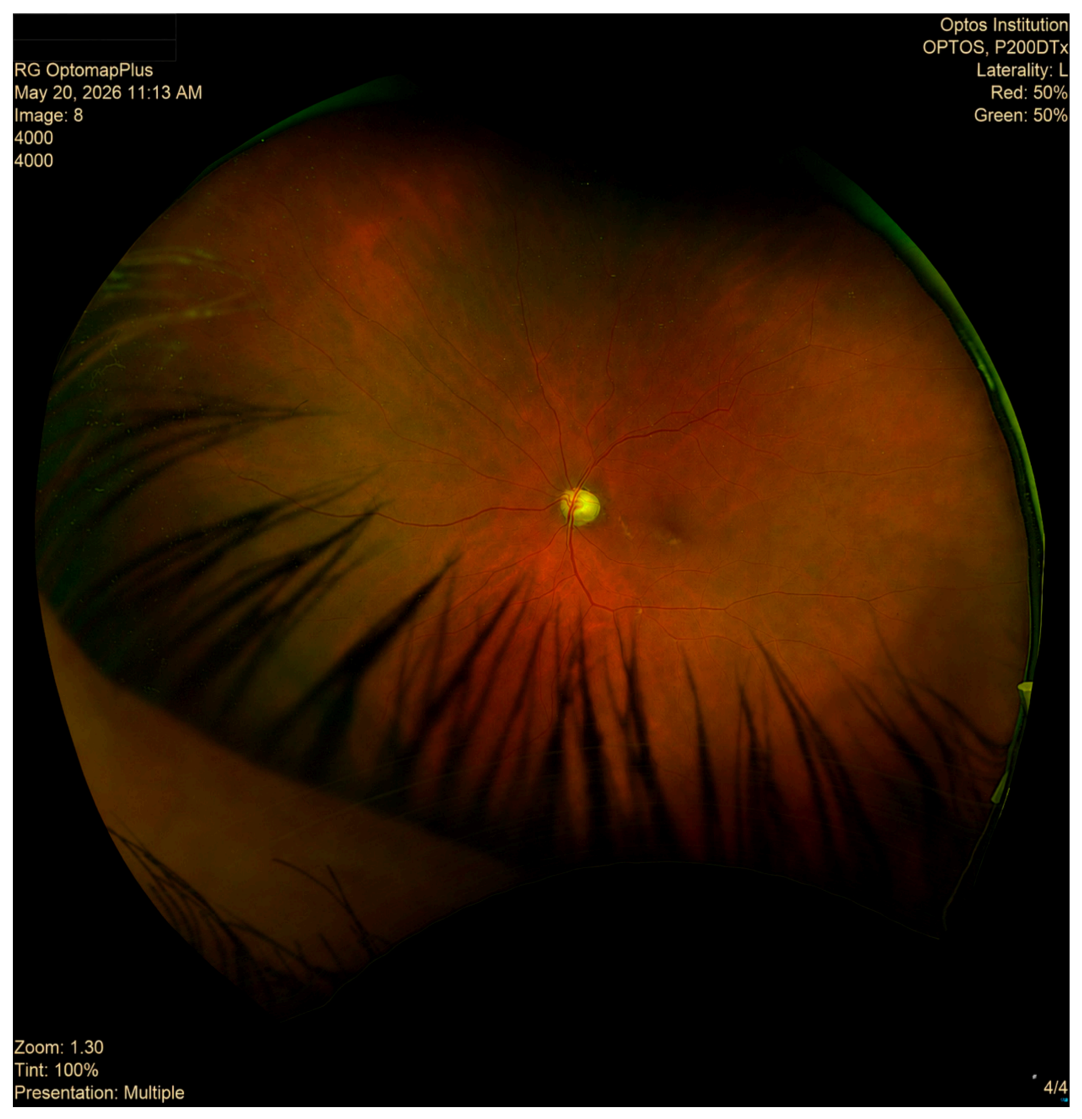
Patient’s fundus photo at 3 years follow-up: LE with a pale disc, atrophy of the pigmentary epithelium in the macular region and marked arteriolar narrowing, turgescent veins and arteriovenous crossing abnormalities.

**Figure 6:**
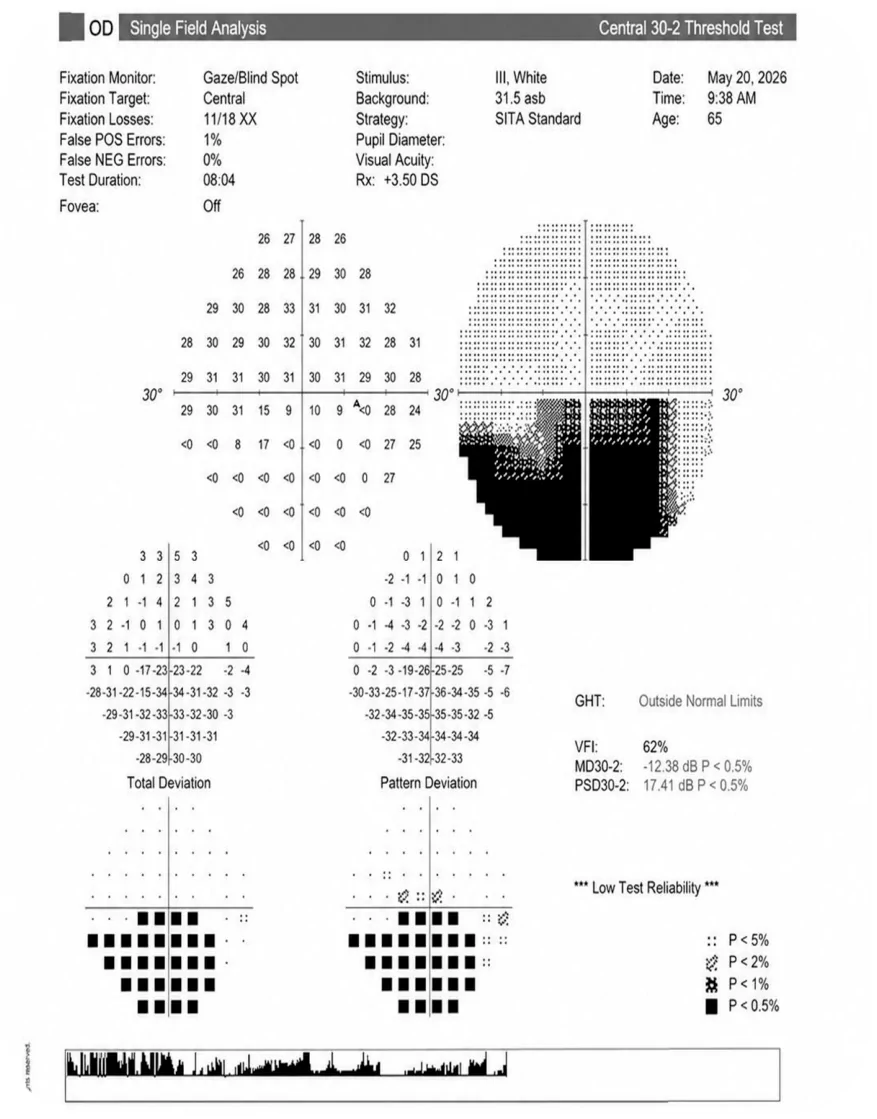
Humphrey VF at 3 years follow-up—RE.

**Figure 7:**
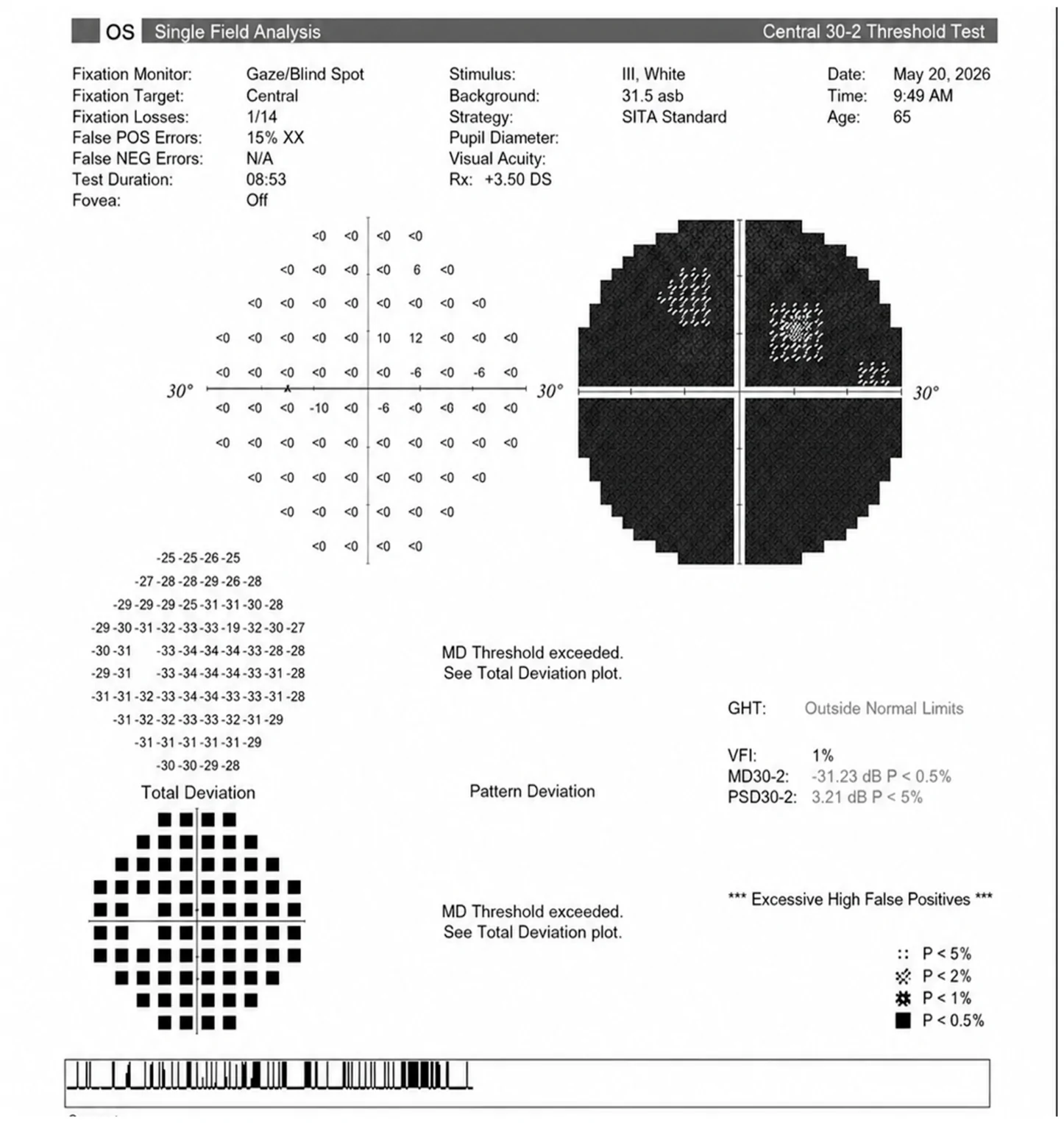
Humphrey VF at 3 years follow-up—LE.

**Figure 8:**
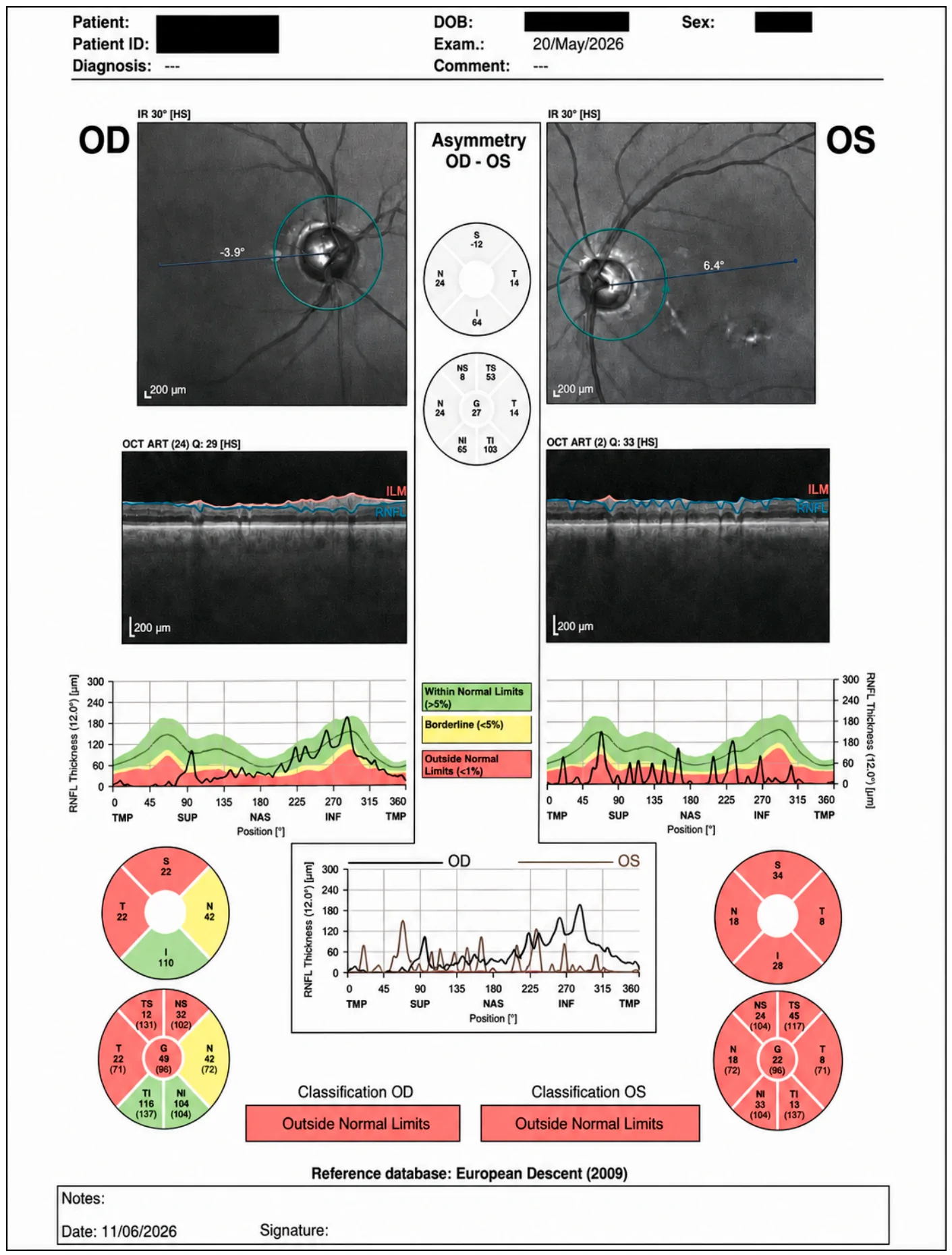
OCT showing BE RNFL thinning, LE > RE.

Three years of follow-up of this patient showed no other major events, neither systemic nor ophthalmological. BCVA was RE 1 Snellen (with inferior altitudinal defect) and LE light perception. Fundus examination revealed RE pale disc in the superior part and marked arteriolar narrowing, turgescent veins and arteriovenous crossing abnormalities (Figure 4). Fundus examination revealed LE pale disc, atrophy of the pigmentary epithelium in the macular region and marked arteriolar narrowing, as well as turgescent veins and arteriovenous crossing abnormalities (Figure 5). The VF was stable, showing inferior altitudinal defect in the RE and an absolute scotoma in the LE (Figure 6 and Figure 7). According to the visual field results, the optic nerve head OCT at 3 years follow-up revealed reduced RNFL thickness in the superior and temporal quadrants of the RE and generalized RNFL thickness reduction in the LE (Figure 8). However, besides all these changes, the patient was independent and had a functional life.

## 3. Discussion

NA-AION is a well-established clinical entity, most frequently with a unilateral presentation [7]. The link between NA-AION and systemic or local predisposing factors has been widely studied. These factors include arterial hypertension, diabetes mellitus, dyslipidemia, obstructive sleep apnea, nocturnal blood pressure fluctuations and underlying atherosclerotic cardiovascular disease [15,20]. Other studies revealed that the most important element in the pathophysiology of this disease is the patient’s crowded optic disc, his own particular conformation [3]. However, this finding is neither necessary nor sufficient to establish the diagnosis of NA-AION, and it may be difficult to assess during the acute phase because optic disc edema can obscure the physiological cup [21]. Smoking is another risk factor for NA-AION reported in the literature and found among our patient’s comorbidities [22]. The physiopathological mechanisms of NA-AION suggest a hypoperfusion of the short posterior ciliary arteries supplying the optic nerve head. This ischemic insult results in compromised axoplasmic transport and optic nerve head edema [23]. Thus, it is probable that ischemia of the optic nerve in NA-AION, especially in bilateral occurrences, is rather caused by the additive and synergistic effects of these vasculopathic risk factors than by a solitary, isolated effect [24].

On the other hand, bilateral NA-AION is a rare event, and identifying the underlying cause represents a significant diagnostic challenge. This entity remains considerably less well characterized than the unilateral form, and the available evidence derives predominantly from isolated case reports and small case series, with only a limited number of larger cohorts [25,26,27]. Published bilateral NA-AION cases have occurred in different clinical settings, including severe hemorrhage, anemia, atherosclerotic disease, dialysis-associated hypotension, and perioperative hemodynamic instability [17,18,28]. Similar to our case, Appiah et al. presented the case of a 78-year-old male also with hypertension, hyperlipidemia, and severe anemia (with an identical hemoglobin of 6.9 g/dL), who developed bilateral NA-AION on the background of an important post-bleeding hypovolemia [17]. Another common risk factor between the two cases is atherosclerotic disease. The authors reported a significant carotid and vertebrobasilar atheromatosis, while in our patient angiographic assessment demonstrated 54% stenosis of the internal carotid arteries, large calcifications, 95% left external iliac artery and 80% left femoral artery stenoses that needed a femoral bypass procedure. The principal difference between the two cases is that the patient in the other study had severe lower gastrointestinal bleeding, while in our patient, blood loss occurred a few days prior, and visual acuity loss appeared after the brachial dissection was repaired, thus, a direct cause could not be established.

The two patients presented by Bansal et al. (2014) shared the same final common pathway as our case, although the route to it differed: systemic hypotension related to recurrent dialysis, as opposed to multiple cardiovascular risk factors associated with cardiovascular surgery in ours. Some nuances, nonetheless, separate their cases. Their second case carried an additional risk factor—a structurally predisposed optic nerve, with an anatomically crowded disc—and this appearance was used, together with the history of hypotension, as positive support for the diagnosis. No comparable morphological description is available for the first patient of Bansal et al., because the bilateral simultaneous disc edema left no interpretable fellow eye. Similar to the latter, in our patient, both eyes had edematous optic discs at presentation, so the ‘disc at risk’ configuration could not be confirmed; optic disc crowding was therefore not used as a diagnostic criterion [18]. Currently, OCT is a valuable technique, providing objective information on the RNFL thickness and comparison to the fellow eye. Studies show that in time, RNFL thickness decreases in 6 to 12 months post NA-AION and remains stable [29]. Also, considering that RNFL thickness corresponds to certain VF areas, OCT findings should be correlated to VF defects and BCVA [30]. In our case, RNFL thickness was increased in both eyes in the acute phase and decreased to atrophy in about 12 months. OCT findings were well correlated with clinical findings, VF defects and visual acuity. Also, from a clinical point of view, the patient perceived the altitudinal defect from the RE. Moreover, OCT angiography seemed to reveal decreased radial peripapillary capillaries in NA-AION associated with generalized vessel reduction, but further studies are needed in this regard [31].

Moreover, bilateral NA-AION, as well as acute ischemic conditions such as thrombotic cerebrovascular or cardiovascular events, have been associated with polymorphisms in genes encoding platelet glycoproteins. This is the case reported by Lim et al., which highlighted the involvement of prothrombotic risk factors in developing bilateral NA-AION, but with a sequential course of events (twelve months gap between the ocular ischemic events) rather than a simultaneous involvement as in our patient [32].

Regarding the perioperative causes of ischemic optic neuropathy (ION), a 2018 review by Roth and Moss summarized the evidence from registries, case–control studies and case series. Perioperative ION was most often reported after cardiac and spinal surgery, but also following head and neck, orthopedic, urologic, gynecologic, and vascular procedures. The posterior form (PION) predominates overall, whereas the anterior form (AION) was reported particularly in cardiac surgery, where a prospective case–control study of cardiopulmonary bypass identified eight cases, all AION. Pre-existing factors associated with the perioperative ION included peripheral vascular disease, diabetes mellitus, hypertension, anemia, blood transfusion, hypotension, older age and male sex, and carotid artery stenosis. Reported intraoperative factors were prolonged anesthesia, massive blood loss, hemodilution, positioning and hypotension [28]. Our patient carried a substantial burden of pre-existing microvascular and macrovascular disease: diabetes mellitus, an established risk factor for non-arteritic AION, and peripheral arterial disease severe enough to require aorto-femoral bypass. The perioperative period should therefore be regarded as having provided the acute hemodynamic trigger—a fall to 90/50 mmHg with a hemoglobin of 6.9 g/dL—acting upon a vascular substrate that was already predisposed.

To our knowledge, no procedure-specific incidence estimates have been published for aorto-femoral bypass or brachial artery repair; moreover, the available studies report ischemic optic neuropathy (ION) without distinguishing anterior from posterior or arteritic from non-arteritic forms. Rates of perioperative visual loss derived from the Nationwide Inpatient Sample place cardiac surgery highest at 8.64 events per 10,000 procedures, followed at some distance by spinal fusion (3.09). An intermediate band comprises hip and femur procedures (1.86), colorectal resection (1.24) and knee replacement (1.08), while cholecystectomy (0.66) and appendectomy (0.12) fall at the low end of the range [33]. However, the available epidemiological data indicate that perioperative ION risk tracks with the hemodynamic burden of the perioperative period rather than with any specific operation. The risk factors identified in these cohorts—advanced age, male sex, transfusion, obesity, prolonged bypass and postoperative anemia—are patient- and hemodynamic-related rather than procedure specific [33]. In this context, the vascular disease burden that led to surgery in our case—diffuse calcified atherosclerosis with high-grade iliac and femoral stenoses, diabetes mellitus and active smoking—is itself a plausible confounder of any apparent procedure-related association. Thus, no causal relationship between the surgical procedures and the optic nerve ischemia can be inferred from this single case.

In order to guide the clinical management and optimize patient outcomes, a multidisciplinary approach is often needed, as well as a whole-body vascular check-up [34,35]. Bansal et al. argued for preventive management for at-risk populations based on 24 h ambulatory blood pressure monitoring to identify nocturnal dipping profile, avoidance of abrupt hypotension during dialysis, and active correction of anemia [18]. AION treatment usually begins with risk factors management and systemic assessment. Even if a curative treatment does not exist, the goal is to prevent fellow eye involvement and the appearance of other potentially fatal events such as cerebral ischemic stroke [7,34]. In line with these considerations, the treatment protocol in our case was the following: in the acute phase, the hypotension was managed by urgent surgical repair of the arterial dissection. Once the patient recovered from this critical event, long-term treatment was directed at secondary vascular prevention and comprised antiplatelet and anticoagulant therapy, antidiabetic treatment, lipid-lowering agents, antihypertensive medication and vasodilators for peripheral arterial disease. Lifestyle measures were also implemented, including smoking cessation and dietary modification together with regular physical activity. Blood pressure control in this setting requires particular care. While antihypertensive treatment was indicated by the patient’s underlying hypertension, the ischemic event itself arose from a fall in perfusion pressure, and avoidance of excessive or nocturnal blood pressure reduction was considered a priority—an approach consistent with the recommendations of Bansal et al., who emphasized the risk of nocturnal dipping and recommended ambulatory monitoring in patients at risk.

The reported visual outcomes were variable, but are frequently poor, particularly when the initial visual loss is profound or the systemic hypoperfusion is prolonged [18]. The first patient of Bansal et al. had a BCVA that deteriorated to hand movements and counting fingers before recovering to approximately 6/9 in both eyes, but without any change in the inferior altitudinal field defect. Their second patient illustrated the opposite pole: presenting with no perception of light bilaterally, he recovered to 6/9.5 in one eye but retained only perception of light in the other [18].

The natural evolution of the disease has been described by the Ischemic Optic Neuropathy Decompression Trial (IONDT): approximately 20% of patients regained 3 or more lines at 2-year follow-up, about 20% lost 3 or more lines at 2-year follow-up, and in the rest of the patients the BCVA remained unchanged [36]. In accordance with this study, our patient’s VF remained unchanged but his BCVA improved at 3 years follow-up.

Unlike previously reported cases in which a single precipitating event was predominant (such as major hemorrhage or dialysis-associated hypotension), no single factor can fully explain the presentation in our patient. Studies have demonstrated that the underlying generalized atherosclerosis, along with diabetes mellitus, dyslipidemia, and smoking, can induce a state of chronic endothelial dysfunction and microangiopathy [37]. These long-standing conditions can lead to a progressive alteration of the microvessels, consequently damaging the intrinsic autoregulatory capacity of the posterior ciliary arteries supplying the optic nerve head [38]. Upon this fragile anatomical baseline, the acute onset of severe anemia and systemic hypotension in our patient acted as critical precipitating factors. However, the much poorer visual outcome in the left eye also suggests that the systemic insult alone may not explain the entire clinical picture and that inter-eye differences in local anatomical or vascular susceptibility may have contributed. As this is a single observational case, the relative contributions of each factor cannot be established, and the proposed synergistic mechanism remains a clinically plausible interpretation rather than a proven causal relationship.

An important limitation of our case is the absence of a histological result via temporal artery biopsy. Temporal artery biopsy is the gold standard in differentiating NA-AION and A-AION [39]. The specificity of the procedure for A-AION can be up to 100%, while the sensitivity is about 77%, but in order to have an accurate result, the biopsy should be about 2–3 cm long [40]. Unfortunately, in our case, the temporal artery biopsy could not be performed due to the patient’s high risk of bleeding and wound-related complications, given his fragile systemic context (severe anemia, hypotension, tachycardia, and a recent history of two major vascular procedures performed in close succession). Thus, the patient’s limited physiological reserve made an additional invasive surgical procedure inappropriate during the acute admission. Even a short biopsy specimen would have still posed an important risk, together with a limited value in excluding the disease compared to the standard procedure. The current recommendations favor obtaining a specimen longer than 1 cm to reduce the high probability of a false-negative result, because of the often-segmental distribution of GCA lesions [41]. Consequently, GCA could not be histologically excluded in our case.

However, an arteritic cause, particularly giant cell arteritis, was specifically considered in our patient’s case. Thus, a targeted medical history was obtained, during which the patient denied headache, jaw claudication, and scalp tenderness. In addition, our patient presented with hyperemic edematous optic discs. This appearance supports a non-arteritic mechanism, in contrast with the characteristic pallid, chalky disc swelling seen in GCA [42]. Moreover, the patient underwent a vessel MRI that did not reveal mural inflammation of the superficial temporal arteries. Recent studies also highlight the importance of the high-resolution MRI in the differential diagnosis between A-AION and NA-AION. It also revealed that bilateral involvement is more frequent than clinically assumed [43,44]. A particularity of this case was the elevated inflammatory markers at presentation. While a high C-reactive protein (CRP) level, along with raised erythrocyte sedimentation rate (ESR) in a patient with signs of anterior ischemic optic neuropathy, are classically associated with an arteritic etiology, such as giant cell arteritis (GCA), in our case these increased levels were interpreted cautiously because the patient had recently undergone two major vascular procedures in close succession, and could therefore have reflected a postoperative inflammatory response. Furthermore, there are studies that point out the possibility of CRP and ESR elevation in the context of such complex surgical interventions, which support our hypothesis [45,46]. In our patient, CRP and ESR values returned to the normal range entirely without immunosuppressive treatment within approximately 1 week, respectively 3 weeks. The kinetics of these markers follows the pattern reported in the postoperative acute-phase response, in which CRP peaks around the third postoperative day and declines thereafter, and is not compatible with untreated active giant cell arteritis in which acute-phase markers remain elevated in the absence of glucocorticoid therapy [45,47]. Moreover, giant cell arteritis diagnosis usually involves more markedly elevated ESR, over 50 mm per hour [48].

Considering the patient’s significant systemic risk profile, the absence of further major cardiovascular or ophthalmological events during the follow-up period is an encouraging clinical observation. However, we cannot definitively attribute this clinical stability to specific interventions based on a single case evolution. The patient understood its condition and risks, changed his lifestyle and diet, quit smoking, was present at every check-up and took his treatment thoroughly. However, because of the potentially profound visual consequences and the persistent uncertainty surrounding its pathophysiological mechanisms, NA-AION requires ongoing attention in clinical practice and medical education, and a comprehensive multidisciplinary approach, particularly from ophthalmologists, cardiologists, neurologists, and primary care physicians. Taking into account that the disease has a vascular determinant, we have to consider the patient as having major cardiovascular risk.

The interest of the present case lies in the convergence of several factors capable of compromising optic nerve head perfusion, including severe anemia, systemic hypotension, associated with extensive atherosclerotic disease in a high-risk vascular patient. In addition, the case illustrates the diagnostic challenge of differentiating a non-arteritic hypoperfusion mechanism from an arteritic process in a recently operated patient with elevated inflammatory markers. The novelty of this report lies in the complexity of the vascular pathology and hemodynamic insult, as well as in the serial documentation of acute-phase markers, which normalized without corticosteroid therapy, in a patient followed for three years without further ischemic events.

### Limitations

Even though we reached a presumptive clinical diagnosis and the MRI was suggestive for NA-AION, the lack of a temporal artery biopsy precludes the absolute exclusion of GCA, and this represents a limitation of our research. However, considering the fact that in three years of follow-up the patient had no other events, the initial diagnosis was probably the right one.

## 4. Conclusions

Bilateral NA-AION is a rare event and is usually associated with a systemic cause. In patients with advanced cardiovascular disease and generalized atherosclerosis, the combination of acute blood loss, severe anemia, and transient systemic hypotension can act synergistically to disrupt optic nerve head perfusion.

Given the significant impact of this disease on patients’ quality of life, it remains a topic of interest for both ophthalmologists and neurologists worldwide. Our experience highlights the potential value of a prompt and comprehensive systemic assessment in high-risk cardiovascular patients. While irreversible visual loss may still occur, this proactive approach may help stabilize the patient and improve overall systemic outcomes. While broad conclusions cannot be drawn from a single case, larger prospective cohort studies are needed to validate our observations.

## Figures and Tables

**Table 1:** Most important blood tests with abnormal results.

Parameter	Value
**Hematology**	
Hemoglobin	6.9 g/dL
Red blood cell count	2.4 × 10^6^/mm^3^
Hematocrit	20.6%
Mean corpuscular volume	92 fL
Platelets	147,000/mm^3^
**Inflammatory markers**	
Erythrocyte sedimentation rate (ESR)	45 mm/h
C-reactive protein (CRP)	59.7 mg/L
**Renal function**	
Serum creatinine	1.52 mg/dL
Urea	87.74 mg/dL
Glomerular Filtration Rate (GFR)	51.17 mL/min/1.73 m^2^
**Metabolic/lipid profile**	
Blood glucose	117 mg/dL
Low-Density Lipoprotein (LDL)	103 mg/dL
**Cardiac markers**	
Troponin I	81.9 pg/mL
**Coagulation**	
Fibrinogen	713 mg/dL
D-dimer	331 ng/mL

## Data Availability

The original contributions presented in this study are included in the article. Further inquiries can be directed to the corresponding authors.

## Abbreviations

The following abbreviations are used in this manuscript:

A-AION	Arteritic anterior ischemic optic neuropathy
AION	Anterior ischemic optic neuropathy
BCVA	Best corrected visual acuity
BE	Both eyes
BUN	Blood urea nitrogen
CRP	C-reactive protein
CT	Computed Tomograph
ESR	Erythrocyte sedimentation rate
GFR	Glomerular Filtration Rate
Hct	Hematocrit
IONDT	Ischemic Optic Neuropathy Decompression Trial
IOP	Intraocular pressure
LE	Left eye
LP	Light perception
MCV	Mean corpuscular volume
MRI	Magnetic Resonance Imaging
NA-AION	Non-arteritic anterior ischemic optic neuropathy
PION	Posterior ischemic optic neuropathy
RAPD	Relative afferent pupillary defect
RE	Right eye
RNFL	Retinal nerve fiber layer
VF	Visual field
